# Safety and Efficacy of Three Variants of Canaloplasty with Phacoemulsification to Treat Open-Angle Glaucoma and Cataract: 12-Month Follow-Up

**DOI:** 10.3390/jcm11216501

**Published:** 2022-11-02

**Authors:** Aleksandra K. Kicińska, Monika E. Danielewska, Marek Rękas

**Affiliations:** 1 Department of Ophthalmology, Military Institute of Medicine, 04-141 Warsaw, Poland; 2 Department of Biomedical Engineering, Faculty of Fundamental Problems of Technology, Wroclaw University of Science and Technology, 50-370 Wroclaw, Poland

**Keywords:** MIGS, mini-invasive antiglaucoma surgery, POAG, canaloplasty, minicanaloplasty

## Abstract

Background: A single-center prospective randomized observational study to compare three types of canaloplasty, i.e., ab externo (ABeC), minicanaloplasty (miniABeC) and ab interno, (ABiC) combined with cataract surgery in primary open-angle glaucoma (POAG) patients over 12 months. Methods: 48 POAG patients underwent one of three canaloplasty procedures: ABeC (16 eyes), miniABeC (16 eyes) or ABiC (16 eyes) or combined with phacoemulsification. Patients were assessed at baseline, at day 0–1–7 and at month 1–3–6–12. Successful treatment was defined as unmedicated IOP reduction ≥20%. Complete surgical success was defined as an IOP ≤ 15 mmHg without medications, and a qualified surgical success as IOP ≤ 15 mmHg with or without medications. Results: Pre-washout IOP median values (mmHg) were 17 (ABeC), 18 (miniABeC) and 17 (AbiC) and decreased at 12-month follow up postoperatively to 13 (*p* = 0.005), 13 (*p* = 0.004) and 14 (*p* = 0.008), respectively—successful treatment was achieved in approximately 100% of patients for ABeC and in 93.8% for both miniABeC and AbiC groups. Preoperatively, the median number of medications was 2.0 (range 1–3) (ABeC), 2.0 (1–3) (miniABeC) and 2.0 (0–4) (ABiC); 12-month post-operatively, all medications were withdrawn except in two patients (followed miniABeC and AbiC). Conclusions: The three variants of canaloplasty significantly reduced IOP and the number of medications in patients with mild to moderate POAG and gave no significant complications.

## 1. Introduction

In recent years, surgical methods to restore natural aqueous outflow systems to treat open angle glaucoma (OAG) have become an alternative to more invasive and risk-carrying penetrating surgeries. Among this group, canaloplasty and its various modifications have been of interest since 2005 when they were first introduced [1]. The concept of canaloplasty was born when adding a flexible microcannula to dilate 360° of Schlemm’s canal by Kearney [2] during viscocanalostomy pioneered by Stegmann [3]. Since then, canaloplasty, performed as a single procedure or combined with phacoemulsification [4,5,6,7,8], has been shown to reduce IOP in patients with OAG in a safe and effective way. In terms of IOP-lowering potential canaloplasty was even described by some authors as comparable to trabeculectomy [9,10]. The surgical technique of canaloplasty is designed to address all aspects of outflow resistance—the trabecular meshwork (TM), Schlemm’s canal and the distal outflow system beginning with the collector channels [2]

Over the years, canaloplasty has gained popularity and evolved into a group of procedures [1]. Various modifications of canaloplasty have been described, including catheterless suture placement [11], using various sutures [12], replacing suture with two Stegmann Canal Expander devices [13] or intubation without viscodilatation with Glaucolight [14]. In 2015, Sunman et al. described a variant of surgery with a deeper scleral excision facilitating the uveoscleral outflow pathway, and thus, intensifying the IOP reducing potential compared to the traditional method [15].

Growing demand for moving towards more sparing procedures caused a natural evolution into ab interno canaloplasty (ABiC) [16]. ABiC belongs to minimally invasive glaucoma surgery and as such requires neither peritomy nor sclerectomy and uses corneal incision to access the Schlemm’s canal under the gonioscopic view. However, the ab interno variant makes the placement of a Prolene suture impossible, which imposes severe limitations on the use of this technique. 

Minicanaloplasty, described by Rekas et al. [17], fills the gap between the traditional procedure, which originated from non-penetrating deep sclerectomy (NPDS), and viscocanalostomy, with its ab interno method. It allows for Schlemm’s canal cannulation without meticulous dissection of the trabeculo-Descemet membrane (TDM)—in this procedure, scleral flaps are only needed for accessing the canal [17]. The initial results proved it to be as successful as traditional canaloplasty and ABiC in terms of IOP reduction with a similar safety profile [18]. Following these observations, the aim of this study was to compare three variants of canaloplasty, i.e., ab externo canaloplasty, minicanaloplasty and ab interno canaloplastyin, in terms of efficacy and safety after 12 months. The role of the scleral lake in canaloplasty and its effect on IOP reduction were also of interest to the investigation.

## 2. Materials and Methods

### 2.1. Study Design

This is a prospective randomized observational clinical study on canaloplasty. The study was performed in accordance with the principles of the Declaration of Helsinki and approved by the Bioethics Committee of the Military Institute of Medicine in Warsaw (decision no. 76/WIM/2015). All patients were over 18 years old and able to understand and provide informed consent. The study was registered at www.clinicaltrials.gov: NCT02908633.

A total of 48 eyes of 48 consecutive patients affected by primary open-angle glaucoma underwent one of three variants of canaloplasty, i.e., ab externo canaloplasty (ABeC), minicanaloplasty (miniC) or ab interno canaloplasty (ABiC), combined with phacoemulsification. Operations were performed at the Ophthalmology Department of the Military Institute of Medicine in Warsaw, Poland, by one surgeon, M.R., between February 2016 and July 2019. Patients were randomized into three groups of 16 by a random sorting algorithm using the maximum allowable 10% deviations in a 1:1 allocation ratio on the day of the surgery. Baseline examination, randomization and postoperative care were carried out by the first author (A.K.K.), who had no interest in selecting one procedure in favor of another. The surgeon was excluded from any evaluation to avoid bias. Study subjects remained blind to treatment assignment throughout the course of the study. Data from all of the patients were reviewed over an extended period of time to obtain a comparable sample size at the 12-month follow-up stage. 

All enrollees underwent a full ophthalmic baseline assessment 30 days before surgery (pre-washout), including: history of glaucoma, medication use, IOP measurement using Goldmann applanation tonometry, corrected distance visual acuity (CDVA) converted to the logarithm of the minimum angle of resolution (logMAR), gonioscopy angle grading according to the Spaeth system, slit-lamp biomicroscopy of the anterior segment and fundus indirect ophthalmoscopy of the optic nerve head, including cup-to-disc ratio, presence of a notch or splinter hemorrhage and peripapillary atrophy. In addition, measurements of central corneal thickness (CCT), axial length (AXL) and keratometric parameters required for the intraocular lens (IOL) calculation were taken. All patients were washed out from antiglaucoma medications throughout a minimum 4-week period prior to surgery. On the day of surgery, IOP was measured and determined as post-washout IOP.

Postoperative follow-up examinations were at days 0, 1 and 7 and months 1, 3, 6 and 12. Postoperative evaluation included IOP measurements, CDVA, slit-lamp examination, gonioscopy and ophthalmic medication reporting. Each time, three IOP measurements were taken and the mean value of the IOP was used for the statistical analysis. Tonometry was always performed between 8 and 10 am.

All adverse events were reported; those within 90 days following surgery were considered early, whereas those after 90 days were counted as late.

Surgical success was analyzed in two categories. The definition of qualified success was based on IOP ≤ 15 mmHg with or without medications, while complete success was defined as IOP ≤ 15 mmHg with no antiglaucoma medications. Additionally, the proportion of eyes at 12 months with unmedicated IOP reduction ≥ 20% compared with post-washout was determined.

### 2.2. Patient Inclusion and Exclusion Criteria

Indications for surgery were: coexisting visually significant cataract and primary open-angle glaucoma (POAG) with progression in visual field (VF) loss despite the use of IOP-lowering medications (one to four active ingredients). Eligible patients presented with ophthalmoscopically detectable glaucomatous optic neuropathy and mild to moderate visual field (VF) loss according to Hodapp–Anderson–Parrish criteria [19] and were previously assigned to combined antiglaucoma and cataract surgery. The post-washout IOP value measured at the day of surgery was required to be 18 mmHg or higher. 

Exclusion criteria were: secondary open-angle or narrow-angle glaucoma, history of ocular trauma or inflammation, previous ocular surgery or laser trabeculoplasty and clinically significant corneal dystrophy. Another excluding factor was a history of untreated IOP over 30 mmHg, based on the knowledge that the collector ostia are collapsed at this level of IOP [20].

Only subjects in whom a full procedure of canaloplasty with 360 degrees catheterization (in ABeC, miniABeC and AbiC) and suture placement (in the case of AbeC and miniABeC) could be performed were included in the study.

### 2.3. Surgical Technique

#### 2.3.1. Canaloplasty

The surgeon followed the traditional ab externo canaloplasty technique, which has been extensively reported in the literature [5,7,8,12,21,22,23,24,25,26]. Scleral flaps were parabolic in shape and their size was 5.0 × 5.0 mm and 4.5 × 4 mm. Once the catheterization of the canal was completed and the distal tip exposed at the ostium, a double 10–0 polypropylene suture was tied to it. As the microcatheter was being withdrawn, a viscoelastic (Healon GV) was injected—once every two clock hours. The deep scleral flap was then excised and the superficial flap was sutured tightly with five 10–0 Nylon sutures in a watertight manner in order to avoid filtering bleb formation.

#### 2.3.2. Minicanaloplasty

Minicanaloplasty is a method proposed by the co-author (M.R.), in which the sizes of both the scleral flaps are modified [17]. The superficial flap dimension was 4.0 × 1.5 mm and the deep flap was 1.0 × 1.0 mm. No TDM was dissected and the deep scleral flap remained unexcised. Sclerectomy was only performed to access Schlemm’s canal. Catheterization and suture placement were performed in an identical fashion to that in ab externo canaloplasty.

#### 2.3.3. Ab Interno Canaloplasty

Ab interno canaloplasty is a method belonging to mini-invasive antiglaucoma surgery (MIGS), and hence, it requires no sclerectomy [27]. This procedure was performed under gonioscopic view. The iTrack device was advanced through the canal’s whole circumference and a viscoelastic was injected during its withdrawal as in ABeC. Prolene suture placement was not possible with this method.

All variants of canaloplasty were combined with cataract phacoemulsification.

### 2.4. Statistical Analysis

The mean values of IOP and CDVA in all patient groups did not follow a normal distribution at specific time stages before and after surgery (Kolmogorov–Smirnov test; *p* < 0.05). Hence, the Wilcoxon signed-rank test was used to test the temporal changes of values of these parameters in each group. Following Armstrong [28], no correction for multiple comparisons was applied. 

To compare the median values of IOP, CDVA and glaucoma medications between the three considered groups of patients at each stage before and after surgery, the Kruskal–Wallis test was used. Additionally, Kaplan–Meier survival analysis and the log-rank test were used to compare the cumulative incidence of qualified and complete success between the considered groups. Fisher's exact independence test was used to compare dependence between the group factor and the proportion of patients who achieved a 20% reduction in IOP. Differences in complications between groups were determined using the Chi-square test.

In all tests, the significance level was set to an α of 0.05. Calculations were performed using SPSS 22.0 (SPSS, Inc., Chicago, IL, USA). 

## 3. Results

All patients in each group met the study inclusion and exclusion criteria and completed the follow-ups over the period of 12 months. One patient was excluded from the study because the post-washout IOP value was below 18 mmHg and one because of incomplete catheterization of Schlemm’s canal. Table 1 shows the patients’ demographic data, and Table 2 presents the results of temporal changes in IOP values in all patient groups together with the results of the Wilcoxon signed-rank tests.

### 3.1. Intraocular Pressure Lowering

**Figure 1:**
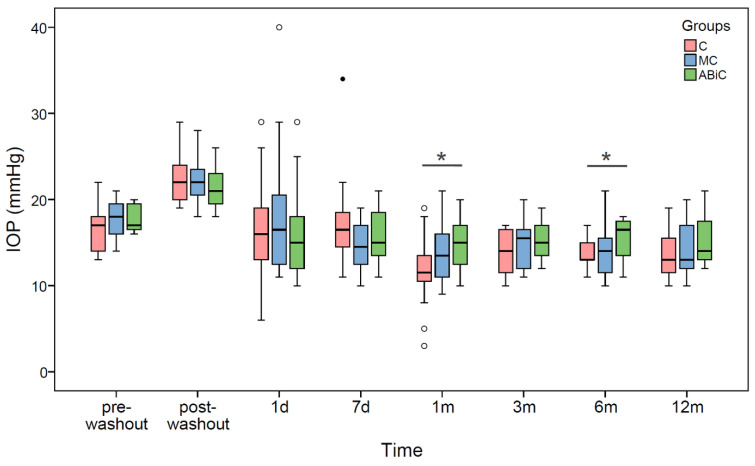
Boxplots of IOP median values at each time stage before and after surgery in three groups of patients: ab externo canaloplasty (ABeC), minicanaloplasty (miniABeC) and ab interno canaloplasty (ABiC). Asterisks denote statistically significant differences between particular groups (*p* < 0.05).

The washout procedure resulted in a statistically significant increase in the IOP median values by 5.0 mmHg, 4.0 mmHg and 4.0 mmHg in the ABeC, miniABeC and ABiC groups, respectively (Wilcoxon test, all *p* ≤ 0.001). At 12 months after surgery, the IOP median values decreased statistically significantly, with respect to both pre- and post-washout stages, in all patient groups (see Table 2 and Figure 1). The same results were observed from 1 month postoperatively in the ABeC group as well as from 7 days postoperatively in the miniABeC and ABiC groups.

### 3.2. Comparison between Groups

There were statistically significant differences in the IOP median values between the ABeC and ABiC groups at the 1-month (*p* = 0.022) and 6-month (*p* = 0.014) follow-ups (Kruskal–Wallis test, all *p* < 0.05; see Figure 1). Based on the results of log-rank test examining the difference in the probability of surgical success between groups, there were no significant differences between the Kaplan–Meier curves of the individual groups (*p* > 0.05) for both complete and qualified success. Application of Fisher's exact independence test showed no significant dependence between the group factor and the proportion of patients who achieved a 20% reduction in intraocular pressure within one year (*df* = 2, *V* = 0.17, *p* = 0.504)

### 3.3. Change in Glaucoma Medication

The median numbers and ranges of medications at all time stages for all groups are presented in Table 3. At the 12-month follow up postoperatively, all medications were withdrawn in in all patients after ABeC and in 15 out of 16 patients in both after miniABeC and ABiC groups (~94%). One patient required addition of one topical medication after 1 month post miniABeC and further intensification of treatment ending with four topical medications at 12 months. A second patient following ABiC was using one medication at the 6-month and three medications at the 12-month follow-up. 

### 3.4. Surgical Success

**Figure 2:**
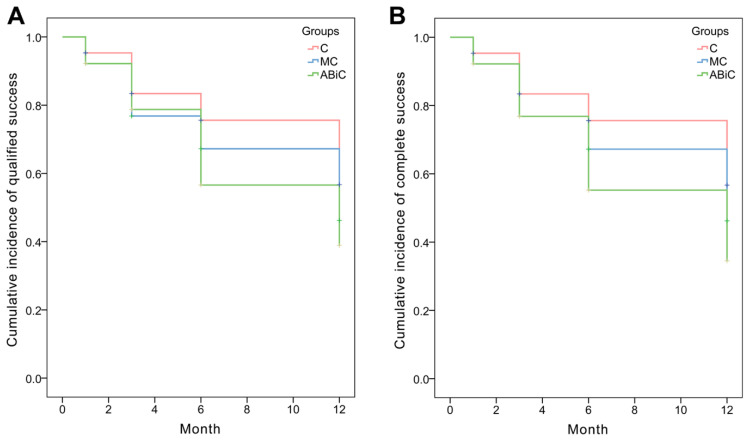
Kaplan–Meier survival analysis: (**A**) qualified success (IOP ≤ 15 mmHg with or without medications) and (**B**) complete success (IOP ≤ 15 mmHg without medications).

Kaplan–Meier cumulative incidence of qualified success was 75.0% (ABeC), 65.6% (miniABeC) and 59.4% (ABiC) (*p* = 0.398) after 12 months, while cumulative incidence of complete success after 12 months of observation was 75.0% (ABeC), 65.6% (miniABeC) and 56.2% (ABiC) (*p* = 0.269; see Figure 2). At the 12-month follow-up, the unmedicated IOP reduction ≥ 20 mmHg was achieved in approximately 100% of patients for ABeC and in 93.8% for miniABeC and ABiC in relation to the post-washout stage.

### 3.5. Visual Acuity Results

**Figure 3:**
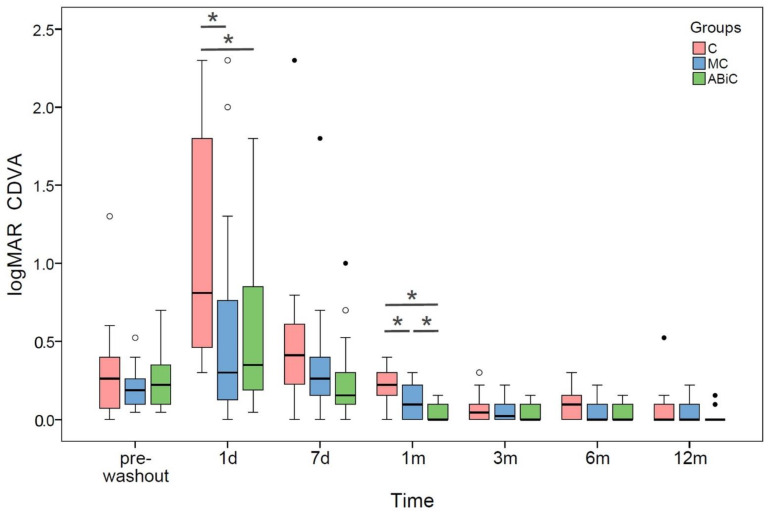
Boxplots of CDVA median values at each time stage before and after surgery in three groups of patients: ab externo canaloplasty (ABeC), minicanaloplasty (miniABeC) and ab interno canaloplasty (ABiC). Asterisks denote statistically significant differences between particular groups (*p* < 0.05).

The median values for CDVA improved statistically significantly from 0.26 to 0.00 logMAR in the ABeC group, from 0.19 to 0.00 logMAR in the miniABeC group, and from 0.22 to 0.00 logMAR in the ABiC group, at 12 months after surgery compared to the pre-washout stage (Wilcoxon test, all *p* ≤ 0.006). The CDVA differed statistically significantly between particular groups at 1 day and 1 month postoperatively (Kruskal–Wallis test, all *p* < 0.05; see Figure 3).

### 3.6. Incidence of Postsurgical Complications

The incidence of complications following all three procedures is shown in Table 4. No intraoperative adverse events were noted. In all variants requiring it, 360° dilation and insertion of a double suture into the Schlemm’s canal was successful. The most common complications were microhyphema—defined as erythrocytes without a layer of blood (gonioscopically confirmed)—and hyphema—defined as layered blood in the anterior chamber. Only one patient required anterior chamber lavage for this reason, following ABeC. A raise in IOP of ≥30 mmHg was noted in all three variants of surgery, and in all cases, this was resolved within 2 weeks with topical antiglaucoma medications. One case of intravitreal hemorrhage occurred, following miniABeC; however, it resolved spontaneously. Two patients suffered from cystic macular edema: one after ABeC and one after miniABeC. Both were successfully treated with topical nepafenac 0.3% and dexamethasone 0.1% for 3 weeks. Interestingly, one patient undergoing ABeC developed a filtering bleb. Its morphology, however, did not resemble filtering blebs post sclerectomy. Additionally, this was the only case of transient hypotony (IOP of 3 mmHg) with a normal anterior chamber. Other complications included transient Descemet’s membrane folds related to the phacoemulsification procedure and corneal erosion.

## 4. Discussion

This prospective randomized trial was conducted to make a contribution to the existing literature on canaloplasty and its various modifications. The trial demonstrated that three variants of canaloplasty, i.e., ab externo, minicanaloplasty and ab interno, combined with cataract surgery provide effective reduction in IOP and medications in Caucasian patients with mild to moderate POAG in a safe way; however, the study was not powered to detect statistically significant changes in IOP. In that sense, our study has a pilot character. Twelve months postoperatively, there were no significant differences between the groups in terms of median IOP reduction and similar rates of effective treatment were observed in all groups, which suggests that all the procedures are effective and may be performed interchangeably. The results of this study are consistent with previously published data on phacocanaloplasty [5,6,10,29], and initial results of minicanaloplasty [17]. This study also shows promising results for ABiC, which was found to be as effective as other variants, and, to the authors’ knowledge, this is the first trial to compare it to traditional canaloplasty in a prospective randomized manner. The amount of data on ABiC in the literature are still insufficient and a longer follow-up can show whether it will be a tissue-sparing alternative to ABeC or miniABeC. However, in our study, two patients following ABiC required readministration of antiglaucoma medication.

Restoring natural outflow pathways in canaloplasty is achieved in three ways. The trabecular tissues are stretched with the use of a tensioning suture in a so-called pilocarpine-like effect. Another way is viscodilating Schlemm’s canal and keeping it open with a suture, which increases its diameter, thus lowering the resistance to flow. Finally, injecting viscoelastic material may open herniations and enable aqueous access to collector channel ostia throughout the whole circumference [21]. Canaloplasty evolved from viscocanalostomy where TDM functions as a site of controlled aqueous outflow [3]. In viscocanalostomy, however, viscodilatation was limited to sclerectomy margins only. This limits the IOP reducing potential of this procedure compared to canaloplasty, as shown by Koerber et al [30]. In NPDS, the inner wall of Schlemm’s canal and the juxtacanalicular TM are peeled off the underlying trabeculum and the superficial flap is loosely attached in order to ease subconjunctival filtration. The aqueous humor that percolates through the TDM collects in the intrascleral space and acts as a reservoir. By contrast, the superficial flap in canaloplasty and viscocanalostomy is sutured tightly to avoid bleb formation and the internal wall of Schlemm’s canal stays untouched. It remains unknown if and to what extent the intrascleral lake supports the IOP-lowering effect of canaloplasty; there are, however, alternate routes of aqueous humor percolation that are theoretically possible, such as transscleral into subconjunctival space. Grieshaber et al. have reported this type of outflow based on the migration of fluorescein dye from Schlemm’s canal into episcleral veins. Mastropasqua et al. [31] supported this thesis by confirming the presence of epithelial microcysts in the bulbar conjunctiva after canaloplasty, similar to those found in filtering blebs after successful trabeculectomy [32,33,34]. These cysts were larger in number at the site of surgery. The authors explain this as occurring because of easier aqueous filtration through reduced scleral thickness after deep flap excision at the site of surgery. This was earlier postulated by Grieshaber et al. who noticed IOP drop caused by goniopuncture without bleb formation, suggesting that TDM plays a role in ABeC [7]. In a study by Byszewska et al., ABeC led to a more effective decrease in IOP than NPDS in a 24-month observation study [29], which, on the other hand, may suggest that restoring natural outflow pathways eventually wins over transscleral percolation. The main idea behind minicanaloplasty was to perform a minimal size sclerectomy only to access Schlemm’s canal and to analyze whether it can compete with the traditional procedure. The 12-month follow-up period does not entitle us to make claims on the importance of the scleral lake; further observation might, however, indicate that its formation is necessary, which implies the need for more invasive procedures. Further observation is crucial in this matter, as one of the NPDS’s long-term mechanisms of action–the presence of new aqueous vessels in the sclera adjacent to the dissection site and suprachoroidal drainage–could also possibly cause traditional canaloplasty to win over minicanaloplasty [35].

The tight closure of the scleral flaps in ABeC is intended to prevent filtering bleb formation. In our study, only one filtering bleb occurred following ABeC. It was more a bleb-like elevation of the conjunctiva similar to the ones described in previously published cases [36]. This was accompanied by IOP drop up to 3 mmHg persisting three months postoperatively. After this period IOP normalized and the bleb was not detectable in as-OCT. IOP measured in this individual at 12 months did not differ much from other patients following ABeC and reached 17 mmHg. The mechanism most probably responsible for bleb formation was TDM microperforation.

Canaloplasty has been of interest not only as an IOP-reducing procedure but also prospectively in antiglaucoma gene therapy. Schlemm’s canal was suggested as a promising site of gene medication delivery which can be reached by catheterization or suture placement during surgery [37]. MiniABeC could be a faster method than the traditional procedure to gain access for TM-targeted gene therapeutics.

The safety profile of canaloplasty in the literature is very high [38]. In this study, none of the serious complications, such as choroidal detachment, retinal detachment, persistent hypotony or endophthalmitis, were observed. Postsurgical complications did not differ significantly between groups. None of the patients required additional glaucoma surgery within the first 12 months postoperatively; however, further observation will bring more information in this regard. The mean CDVA was significantly improved in the majority of cases and vision deterioration happened in only three individuals. This was mainly caused by simultaneous cataract phacoemulsification. The CDVA differed statistically significantly between particular groups at 1 day and 1 month postoperatively, which was probably due to differences in the amount of blood in the anterior chamber. The most frequent and only statistically significant complications in the early postoperative period were microhyphema/hyphema. Other common complications were Descemet folds, inflammation and transient IOP spikes. Presence of blood reflux is generally considered a good prognostic factor [39]; however, the fact that one of the patients after ABeC required surgical lavage is of note. 

Another issue is the effect of phacoemulsification itself on post-surgical drop in IOP (in our study all eyes underwent a combined phacocanaloplasty procedure). According to different studies, it is on average: 1.4 mmHg, 1.9 mmHg, 1.55 mmHg, 1.88 mmHg, 2.9 mmHg, 3.1 mmHg and 4.9–5.3 mmHg [40,41,42,43,44]. Depending on the type of glaucoma, the largest decline in IOP is observed in eyes with angle-closure glaucoma, and in pseudoexfoliative glaucoma (this effect is transient and after a year IOP gradually increases). From an analysis of the published studies, it can be concluded that the largest decrease in intraocular pressure occurs between the third and sixth month after surgery [45,46]. From our previous studies, this effect remained at a similar level throughout the entire follow-up period which was 1 year, with the lowest values generally occurring after half a year of follow-up [47]. Hayashi et al. described an analogous IOP drop of 6.9 mmHg within 12 months after phacoemulsification, and even greater, as much as 7.2 mmHg, during 24 months after surgery. Generally, it can be concluded that this effect is most strongly expressed during the first year after surgery [48], although there are reports in the literature that a reduction in IOP was noted even 10 years postoperatively [49].

According to the data from the above-mentioned studies [40,41,42,43,44,45,46,47,48,49,50], the conclusion might be drawn that the decrease in IOP after surgery shows a strong inverse correlation with the preoperative depth of the anterior chamber, the width of the anterior chamber angle and the initial level of IOP.

Despite the promising outcome, the results of this study need to be interpreted with caution due to certain study limitations: a relatively small sample size and short follow-up period. Due to relatively small count of patients in the study, we performed a post-hoc estimation of statistical power. We have conducted our analysis according to Schoenfeld's formula for hazard ratio 0.7 at a statistical power of 80% a significance level of 5%. The required group sample should be n = 123. For the aforementioned reasons, it is stressed that data from this trial are of a preliminary nature. Furthermore, because the surgeon involved had many years of experience in non-penetrating and mini-invasive glaucoma surgery preparation in order to perform TDM in ABeC or mini-flaps in miniABeC did not require a long learning curve.

The main strength of the study is that it is the first to the authors’ knowledge to compare different variants of canaloplasty as a treatment for POAG in a randomized prospective manner. The study demonstrates that all three variants of canaloplasty: ABeC, miniABeC and ABiC can be efficient in reducing IOP in mild to moderate POAG and are of a similar safety profile.

## 5. Conclusions

The aim of the study was to compare three variants of canaloplasty. All three variants occurred to result in effective IOP and medications’ number reduction with mild complications. The results also did not differ significantly between groups. The authors conclude that canaloplasty combined with phacoemulsification is a procedure suitable for mild to moderate POAG patients with concomitant cataract, no previous damage to iridocorneal angle and assumed patent outflow system. Novel microsurgical variants of the procedure are unlikely to replace the conventional one; however, they provide alternatives for patients with early indication for surgical intervention.

## 6. Value Statement

What was known:

- ABeCy is a safe and effective technique to treat POAG with an IOP-reducing potential comparable with filtering surgeries.

What this paper adds:

- The study demonstrates that all three variants of canaloplasty, i.e., ABeC, miniABeC and AbiC, can be efficient in reducing IOP in mild to moderate POAG and are of a similar safety profile.
- Avoiding dissection of the TDM, as in miniABeC, may not affect IOP reduction, which questions the importance of the scleral lake in Schlemm’s canal surgery.

## Figures and Tables

**Table 1:** Patients’ demographics. Ab externo canaloplasty (ABeC), minicanaloplasty (miniABeC) and ab interno canaloplasty (ABiC).

Demographic.	ABeC	miniABeC	ABiC
General			
Patients, *n* (%)	16 (33)	16 (33)	16 (33)
Sex, *n* (%)			
Female	12 (75)	10 (62.5)	14 (87.5)
Male	4 (25)	6 (37.5)	2 (12.5)
Age (y)			
Mean ± SD	77 ± 7	74 ± 8	74 ± 7
Range	62–88	61–89	64–81
Eye			
Right, *n* (%)	6 (37.5)	6 (37.5)	7 (43.8)
Left, *n* (%)	10 (62.5)	10 (62.5)	9 (56.2)
Ethnicity, *n* (%)			
Caucasian	16 (100)	16 (100)	16 (100)
Glaucoma characteristics Glaucoma type			
POAG (primary open-angle glaucoma)	16 (100)	16 (100)	16 (100)
MD, Mean ± SD	4.0 ± 2.5	4.0 ± 2.5	4.0 ± 2.5
Drugs, median (range)	2.0 (1–3)	2.0 (1–3)	2.0 (1–3)

**Table 2:** First quartiles (Q1), medians, third quartiles (Q3) and interquartile ranges (IQRs) of IOP at each time stage before and after surgery in three groups of patients: ab externo canaloplasty (ABeC), minicanaloplasty (miniABeC) and ab interno canaloplasty (ABiC). *p* value * and *p* value ** correspond to the results of the Wilcoxon signed-rank tests between particular study stages and the pre-washout or post-washout stages, respectively. All patients in each group completed a 12-month follow-up. Italicized values indicate *p* < 0.05.

	ABeC			miniABeC			ABiC		
Time	IOP (intraocular pressure) (mmHg) Q1, Median, Q3, IQR	*p* Value *	*p* Value **	IOP (mmHg) Q1, Median, Q3, IQR	*p* Value *	*p* Value **	IOP (mmHg) Q1, Median, Q3, IQR	*p* Value *	*p* Value **
pre-washout	14.0, 17.0, 18.0, 4.0	-	*<0.001*	16.0, 18.0, 19.8, 3.8	-	*0.001*	16.3, 17.0, 19.8, 3.5	-	*0.001*
post-washout	20.0, 22.0, 24.0, 4.0	*<0.001*	-	20.3, 22.0, 23.8, 3.5	*0.001*	-	19.3, 21.0, 23.0, 3.7	*0.001*	-
1 d	12.0, 16.0, 19.0, 7.0	0.925	*0.008*	12.3, 16.5, 20.8, 8.5	0.795	*0.031*	12.0, 15.0, 18.0, 6.0	0.088	*0.003*
7 d	14.3, 16.5, 18.8, 4.5	0.705	*0.007*	12.3, 14.5, 17.0, 4.7	*0.007*	*<0.001*	13.3, 15.0, 18.8, 5.5	*0.030*	*<0.001*
1 m	10.3, 11.5, 13.8, 3.5	*0.004*	*<0.001*	11.0, 13.5, 16.0, 5.0	*0.002*	*<0.001*	12.3, 15.0, 17.0, 4.7	*0.003*	*<0.001*
3 m	11.3, 14.0, 16.8, 5.5	*0.016*	*<0.001*	12.0, 15.5, 16.8, 4.8	*0.006*	*0.001*	13.3, 15.0, 17.0, 3.7	*0.001*	*<0.001*
6 m	13.0, 13.0, 15.0, 2.0	*0.003*	*<0.001*	11.3, 14.0, 15.8, 4.5	*0.006*	*<0.001*	13.3, 16.5, 17.8, 4.5	*0.006*	*<0.001*
12 m	11.3, 13.0, 16.3, 5.0	*0.005*	*<0.001*	12.0, 13.0, 17.0, 5.0	*0.004*	*<0.001*	13.0, 14.0, 17.8, 4.8	*0.008*	*<0.001*

**Table 3:** First quartiles (Q1), medians, third quartiles (Q3) and interquartile ranges (IQRs) of CDVA and number of medications (*n*) at each time stage before and after surgery in three groups of patients: ab externo canaloplasty (ABeC), minicanaloplasty (miniABeC) and ab interno canaloplasty (ABiC). *p* value * corresponds to the results of the Wilcoxon signed-rank tests for CDVA between particular study stages and the pre-washout stage. All patients in each group completed a 12-month follow-up. Italicized values indicate *p* < 0.05.

	ABeC			miniABeC			ABiC		
Time	Medications (*n*) Median (Range)	CDVA (logMAR) Q1, Median, Q3, IQR	*p* Value *	Medications (*n*) Median (Range)	CDVA (logMAR) Q1, Median, Q3, IQR	*p* Value *	Medications (*n*) Median (Range)	CDVA (logMAR) Q1, Median, Q3, IQR	*p* Value *
pre-washout	2.0 (1 to 3)	0.06, 0.26, 0.40, 0.34	-	2.0 (1 to 3)	0.10, 0.19, 0.28, 0.18	-	2.0 (0 to 4)	0.10, 0.22, 0.37, 0.27	-
post-washout	0	-	-	0	-	-	0	-	-
1 d	0	0.43, 0.81, 2.05, 1.62	*0.002*	0	0.11, 0.30, 0.88, 0.77	*0.038*	0	0.17, 0.35, 0.92, 0.75	0.069
7 d	0	0.19, 0.41, 0.66, 0.47	0.109	0	0.15, 0.26, 0.40, 0.25	0.272	0	0.10, 0.15, 0.30, 0.20	0.691
1 m	0	0.15, 0.22, 0.30, 0.15	0.637	0	0.00, 0.10, 0.22, 0.22	0.105	0	0.00, 0.00, 0.10, 0.10	*0.001*
3 m	0	0.00, 0.05, 0.10, 0.10	*0.001*	0 (0 to 1)	0.00, 0.02, 0.10, 0.10	*0.001*	0 (0 to 1)	0.00, 0.00, 0.10, 0.10	*0.001*
6 m	0	0.00, 0.10, 0.16, 0.16	*0.003*	0 (0 to 2)	0.00, 0.00, 0.10, 0.10	*0.002*	0 (0 to 1)	0.00, 0.00, 0.10, 0.10	*0.001*
12 m	0	0.00, 0.00, 0.10, 0.10	*0.004*	0 (0 to 4)	0.00, 0.00, 0.10, 0.10	*0.006*	0 (0 to 3)	0.00, 0.00, 0.00, 0.00	*0.001*

**Table 4:** Evaluation of surgical and postsurgical complications with the aid of the Chi-square test. Italicized values indicate *p* < 0.05.

Complications	ABeC	miniABeC	ABiC	*p* Value
Early postoperative				
Elevated IOP (intraocular pressure) (≥30 mmHg)	2/16	1/16	2/16	0.800
Hyphema	8/16	3/16	1/16	*0.013*
Microhyphema	5/16	5/16	11/16	*0.047*
Fibrous strands	1/16	0/16	0/16	0.360
Cystic macular edema	1/16	1/16	0/16	0.593
Vitreous hemorrhage	0/16	1/16	0/16	0.360
Descemet folds	2/16	2/16	6/16	0.133
Corneal erosion	0/16	0/16	1/16	0.360
Bleb formation	0/16	1/16	0/16	0.360
Hypotony (IOP ≤ 5 mmHg)	0/0	1/16	0/0	0.360
